# Management of severe intraoperative hemorrhage during intraventricular neuroendoscopic procedures: the dry field technique

**DOI:** 10.1007/s00701-022-05207-9

**Published:** 2022-04-22

**Authors:** Joachim Oertel, Stefan Linsler, Lea Strohm, Sebastian Senger

**Affiliations:** 1 Klinik für Neurochirurgie, Universität des Saarlandes, Homburg, Saar, Germany; 2 Universität des Saarlandes, Kirrberger Straße 1, Gebäude 90.5, 66424 Homburg, Germany

**Keywords:** Neuroendoscopy, Small chamber technique, Dry field technique, Intraventricular hemorrhage, Complication management

## Abstract

**Objective:**

Neuroendoscopic procedures inside the ventricular system always bear the risk for an unexpected intraoperative hemorrhage with potentially devastating consequences. The authors present here their experience, and a stage-to-stage guide for the endoscopic management of intraoperative hemorrhages.

**Methods:**

A step-by-step guide for the management to gain control of and stop the bleeding is described including a grading system. More advanced techniques are presented in cases examples.

**Conclusion:**

Most of intraoperative hemorrhages can be controlled by constant irrigation and coagulation. More advanced techniques can be applied quickly and easily to ensure control of the hemorrhages and avoid the need for a microsurgical conversion.

**Supplementary Information:**

The online version contains supplementary material available at 10.1007/s00701-022-05207-9.

## Background

The acceptance and knowledge about intraventricular neuroendoscopic procedures have spread over the last decades. Procedures like endoscopic ventriculostomy have become standard in many departments [5, 7, 8], but also, more sophisticated endoscopic procedures such as tumor resection or cyst wall fenestration are more frequently performed due to comparably low complication rates [2]. One of the most feared potentially live threatening complications is an intraoperative hemorrhage. The “red out” leads to an instant loss of vision and orientation of the surgeon. This might cause damage to the surrounding tissue and might result in an abortion of the procedure or a conversion to a microsurgical technique [9]. Although the reports and rates of intraoperative hemorrhage differ in the literature regarding the bleeding rates and severity, the intraoperative managing of this complication is mandatory to every neuroendoscopist [1, 4]. Therefore, the authors present a grading system for intraoperative hemorrhage during endoscopic procedures and a step-by-step escalation of the available surgical techniques to regain intraoperative orientation and to control and stop the hemorrhage.

## Relevant surgical anatomy

**Fig. 1:**
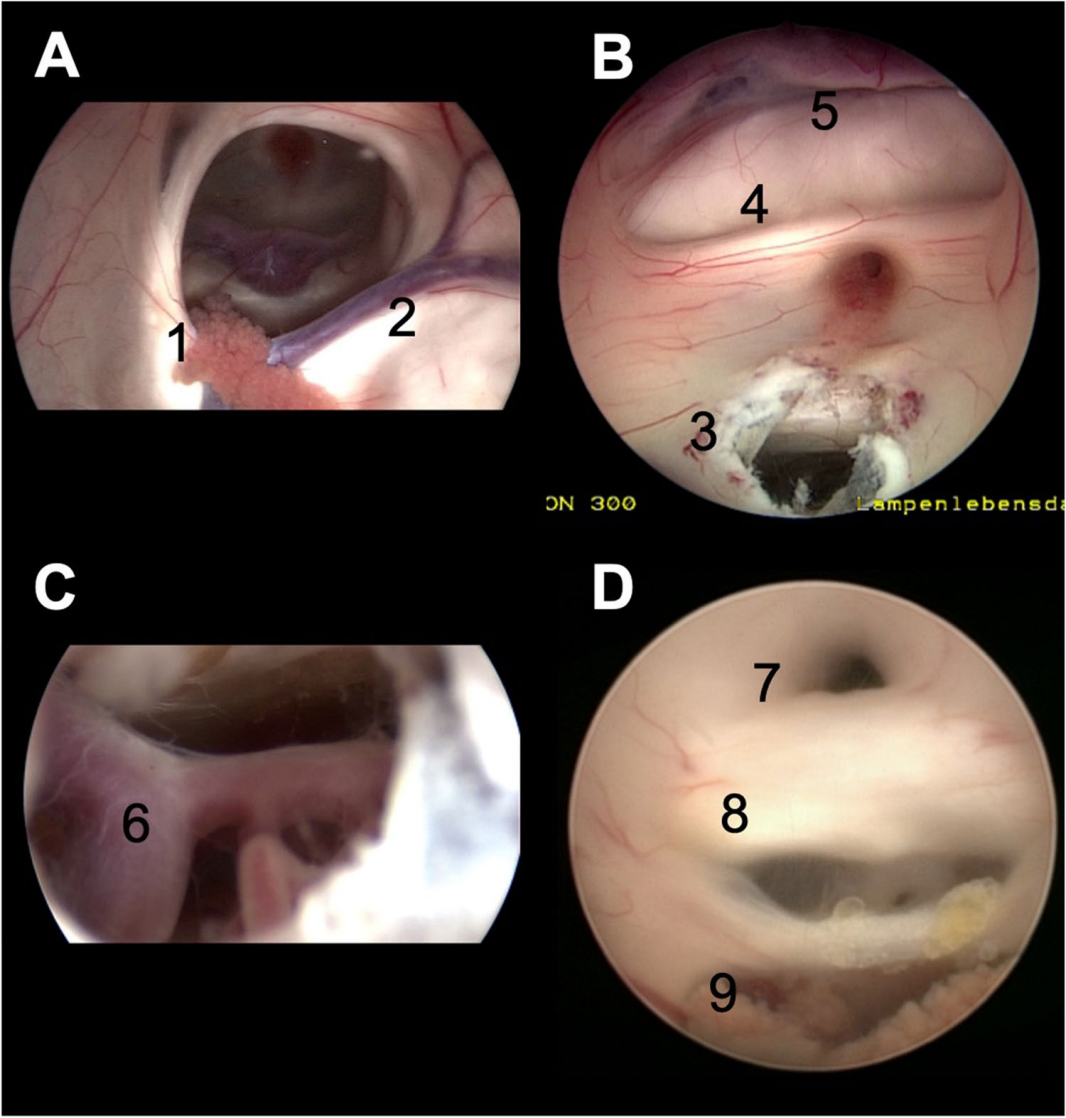
Summary of the intraventricular anatomy. **A** Visualization of the right side ventricle. The choroid plexus (1) and the thalamostriate vein (2) lead to the foramen of Monro in the centre. **B** View of the anterior part of the third ventricle after an ETV stoma (3). The optic chiasm (4), the lamina terminalis and the anterior cerebral arteries (5) can be identified. **C** View through the fenestration at the floor of the third ventricle with the basilar artery (6). **D** View of the posterior part of the third ventricle with the aqueduct (7), posterior commissura (8), and the choroid plexus at the roof (9)

The endoscopic anatomy of the ventricular system has been described in detail many times [10]. Some anatomical structures should be considered for a higher risk for an intraoperative hemorrhage (Fig. 1). The major artery, that is associated with the ventricular system, is the basilar artery. It is under potential risk at the floor of the third ventricle e.g. in case of an ETV. A less common procedure might be the fenestration of the lamina terminals, where the anterior cerebral arteries can be exposed. Certain draining veins should be kept in mind, too. The thalamostriate, the superior choroidal and septal veins meet at the foramen of Monro to form the internal cerebral vein. Any manipulation around these veins such as entering the third ventricle through the foramen of Monro or performing a septostomy can harm these vessels. The chorioid plexus is a well-vascularized structure; it is present in the lateral ventricles, the roof of the third ventricle and as well in the fourth ventricle. Finally, every intra- or paraventricular tumor or tumor like lesion might be well vascularized and can bear the risk of an intraoperative hemorrhage. Tumors and cyst might be stiff or adhesive to adjacent tissue or vessels. This can result in major bleedings during the removal e.g. in colloid cyst removal at the roof of the third ventricle. Therefore, a detailed preoperative imaging including contrast agent MRI and a detailed surgical planning are strongly recommended.

## Hemorrhage grading

**Fig. 2:**
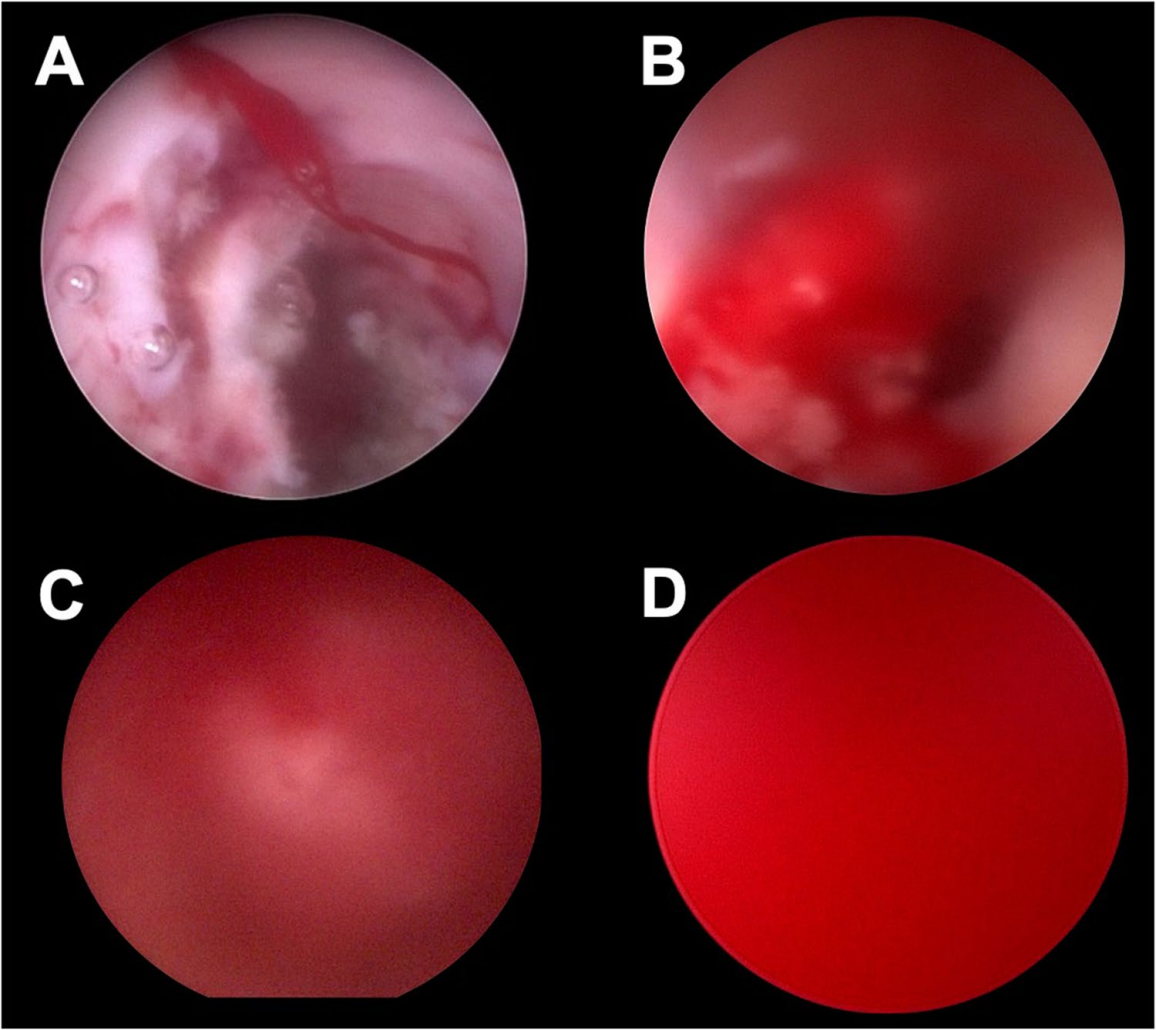
Summary of the hemorrhage grading with grade I (**A**), grade II (**B**), grade III (**C**), and grade IV (**D**)

The intraoperative bleeding in our department is defined by a so far unpublished grading system (Fig. 2). Grade 0 means no kind of hemorrhage. A grade I bleeding is present, when the intraventricular vision is affected by small amounts of blood. A blurry vision occurs that can be easily managed by irrigation. A grade II hemorrhage is defined as a hemorrhage with quickly expanding blood that causes a blurry vision. Blood streaks are identified. The anatomy of the ventricle is still identifiable and the vision can be cleared by irrigation. The grade III hemorrhage is already a major bleeding which significantly affects the endoscopic vision. Anatomical structures can be rarely visualized although constant irrigation is applied. A grade IV hemorrhage describes the so-called red out with complete loss of vision. No anatomical structures can be identified with constant irrigation.

## Perioperative management of intraventricular endoscopic procedures

General recommendation for the save performance of an intraventricular neuroendoscopic procedure have been described manifold in the literature. The equipment depends on the intended procedure and may vary from flexible endoscopes to work sheath based rigid endoscopes. In general speaking, an ideal trajectory planning, anatomical knowledge, and the availability of a neuronavigation system are recommended. All patients should be scheduled for an intensive care unit surveillance. A perioperative antibiotic prophylaxis is mandatory. All patients should receive a postoperative CT scan after 24 h or in case of new neurological deficits. Fever might occur within 48 h after plentiful irrigation. A CSF diagnostic and antibiotic therapy should be initiated if an infection could be assumed. The need for an additional shunt therapy in the follow-up should be kept in mind.

## Step-by-step management of hemorrhages

The irrigation system should be checked and is absolutely vital during the procedure. Irrigation is performed with 35° warm Ringer solution in general. The irrigation outflow at the endoscope should be checked routinely to avoid an unintended ICP increase during the procedure. A clear communication between all involved medical staff in the OR should be obtained. This ensures a short reaction time, and irrigation will be applied as the neurosurgeon intends.

The first step, that is mandatory during an unintended bleeding event, is to obtain the recent position with the endoscope. It is absolute important to fix the recent position independently from the use of a holding system or application of the free hand technique. Otherwise, the orientation within the ventricular system can be lost due to the worsening view. Moving the endoscope without clear visualization and orientation may harm the adjacent tissue and even increase the bleeding.

The second step is to ensure irrigation by the assistant or by an automated device. In grade I or grade II bleedings, the vision should normalize and the bleeding source can be identified. Smaller bleedings can stop spontaneously under constant irrigation. Irrigation can also help to improve the vision in a grade III hemorrhage to identify the anatomic structures again and to find the bleeding source.

**Fig. 3:**
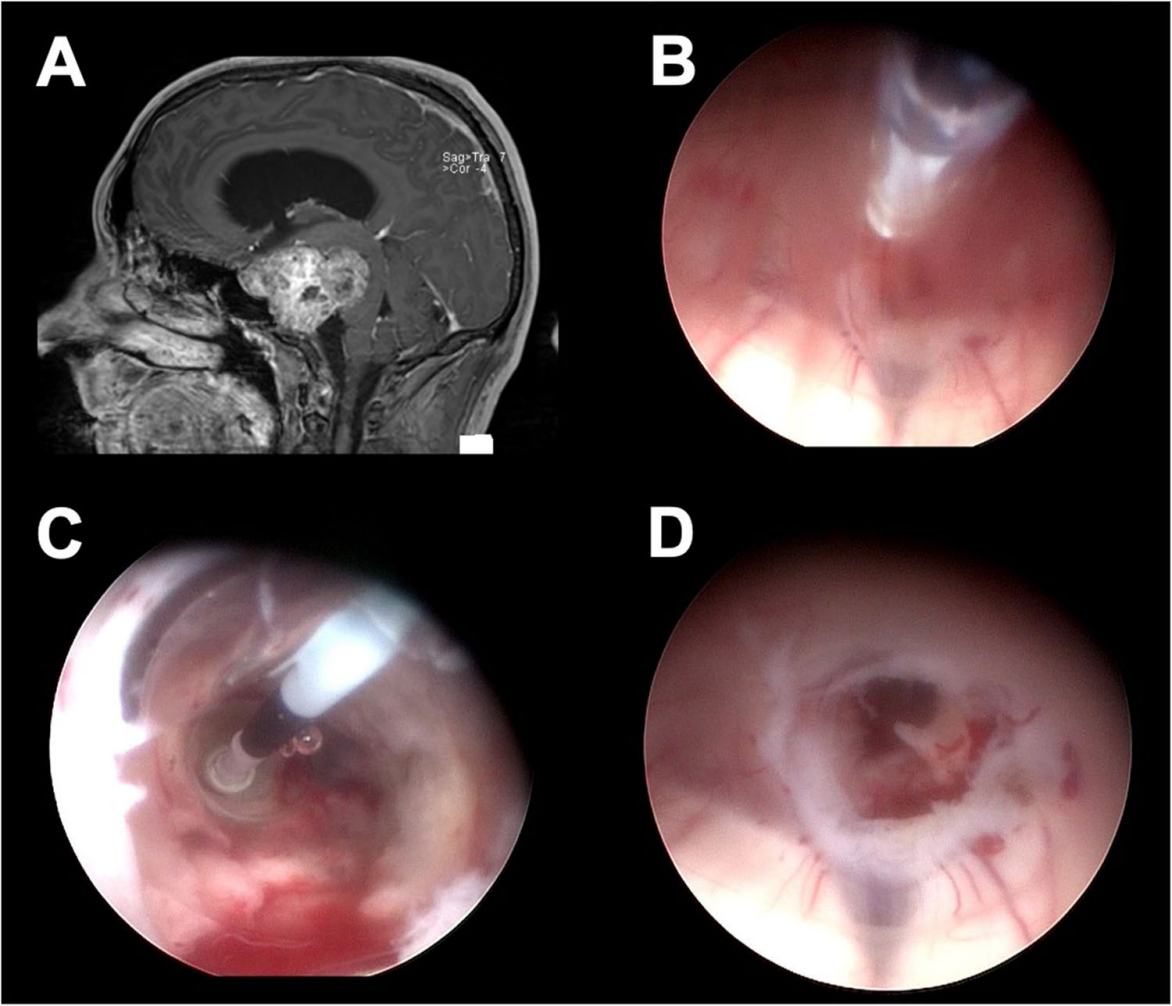
Example of the application of the compression technique. Preoperative MRI of a 42-year-old patient with clivus chordoma (**A**). The patient was scheduled for an ETV due to obstructive hydrocephalus and biopsy. A hemorrhage occurred at the stoma edge during the ETV (**B**). The balloon catheter was reintroduced and dilated for a short amount of time (**C**). The hemorrhage stopped and the surgeon was able to proceed with the tumor biopsy (**D**)

The next step should be the coagulation of the bleeding source, if the hemorrhage does not stop under irrigation. This can be performed with bipolar coagulation electrodes or lasers. In the case of a stoma-associated bleeding, a mild dilation of the balloon catheter may stop the bleeding by compression (Fig. 3). The potential thermic damage to the surrounding tissue must keep in mind.

A further escalation is necessary, if these steps are not sufficient enough to control the hemorrhage and the vision is still insufficient. There are different techniques that could be applied. A slight increase in ICP by closing the outflow channel of the endoscope might stop venous bleedings. This technique has described from experts and requires a detailed monitoring of the patient. However, this technique is not applied in our institute.

**Fig. 4:**
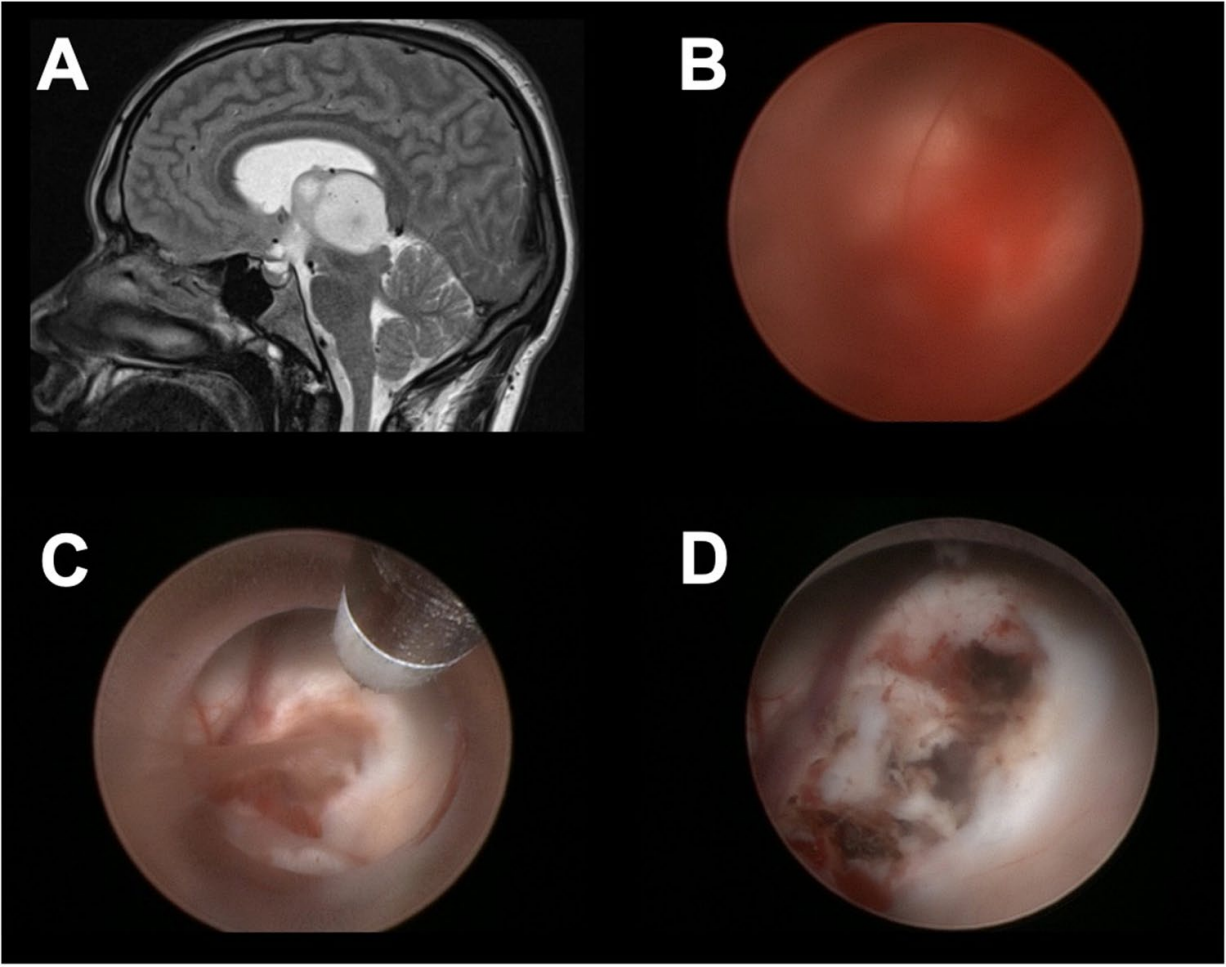
Example of the application of the small chamber technique. Preoperative MRI of a 18-year patient with a glioblastoma (**A**). A grade IV hemorrhage occurred during the tumor biopsy and lasted for 11 min. Irrigation did not improve the vision and no identification of the bleeding source was possible (**B**). The small chamber technique was applied to obtain a clear vision and identification of the injured vessel. The bipolar electrode was used for coagulation (**C**). The hemorrhage stopped and the procedure was finished including an additional ETV (**D**)

Another method is the so-called small chamber technique, which was described by Manwaring et al. first [6]. Trocar-based endoscopes allow a retreat of the endoscope within the working sheath. This creates a small chamber, which can be constantly irrigated and ensures clear vision in a limited area. The endoscope can be placed over the bleeding source and coagulation can be performed under clear vision (Fig. 4).

**Fig. 5:**
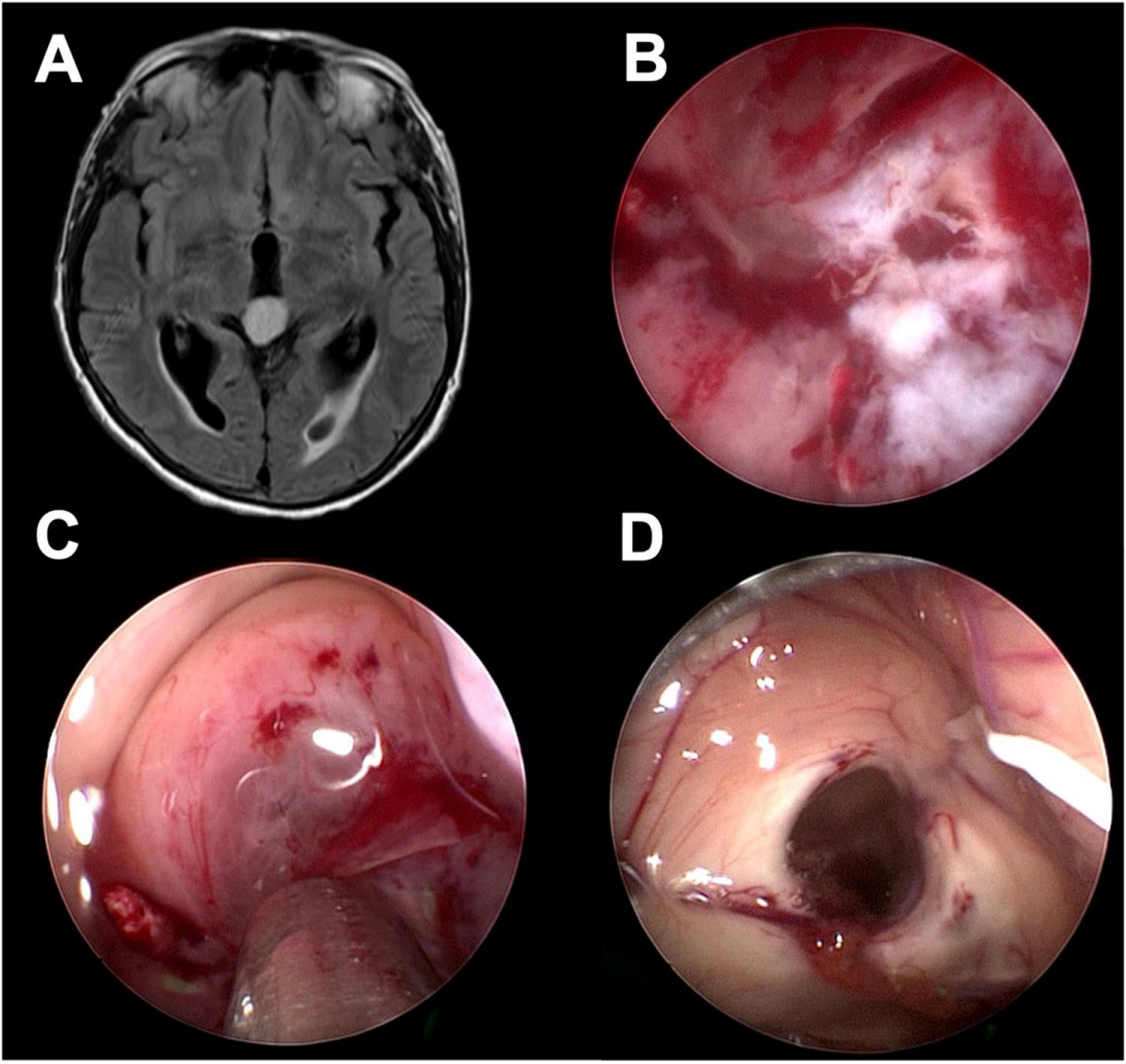
Example of the application of the dry field technique. Preoperative MRI of a 74-year-old patient with anaplastic astrocytoma at the pineal region (**A**). The patient was scheduled for an ETV, tumor biopsy and optional partial resection. The tumor was well vascularized and the surgeon decided to proceed with the procedure under dry field condition (**B**). A partial resection was performed using a endoscopic ultrasound aspirator (**C**). The inspection of the ventricle at the end of the procedure showed no ventricle collapse (**D**). The ventricle system was refilled and no postoperative complications occurred

The more often used technique in our department is the so-called dry field technique [9]. In this technique, the CSF is carefully sucked out using a pediatric sucking tube from the anesthesiologists. This creates an air environment, which has two positive effects. The vision is cleared so the bleeding source and orientation is granted. The gravity and air support the clot formation and supports the hemorrhage management. The ventricles are refilled with ringer solution afterwards. This technique can also be performed in a partial DFT. This means that, if the target of the surgery is placed on the highest position, only parts of the CSF have to be sucked out (Fig. 5).

When the hemorrhage is under control, certain considerations should be kept in mind after the end of the procedure. The third ventricle and aqueduct should be inspected to ensure no blockade by any clot formation. In case of a not removable clot, an ETV is recommended. Alternatively, an EVD should be placed [3, 9].

## Limitations

A potential side effect of the DFT might be the ventricle collapse or a postoperative subdural hematoma or hygroma. In our own case series, none of these effects occurred; however, slow CSF aspiration is mandatory [9]. Uncontrollable bleedings that are not manageable with endoscopic techniques require the conversion to a microsurgical approach [3]. The surgeon and staff should always be prepared for this option, although it might cause further damage to the brain.

## Specific patient information

In general, tumor-associated intraventricular endoscopic procedures have a risk for procedure associated morbidity and mortality of around 10% and 1% respectively [1]. Intraoperative hemorrhage occurred in more than 80% of our own cases; most of them were of grade I and II. Therefore, we advise to inform patients about the common observation of intraoperative hemorrhage prior to surgery. However, most of them can be managed with endoscopic techniques. The rate of conversation to a microsurgical approach is less than 5%. An additional EVD might be necessary. Postoperative fever is a common symptom after intraventricular endoscopy especially under extended irrigation. All patients should be informed that a secondary shunt dependency could occur. This might happened due to the tumor progress, ETV failure, or malresorptive hydrocephalus. The shunt dependency rate in our case series was 11% due to different reasons.

## Summary

1. The endoscopic technique can be a helpful tool in selected cases of intraventricular tumors. Particularly in cases selected for biopsy in intraventricular tumors and in cases located mainly in the third ventricle with difficult access the endoscopic technique might offer distinct advantages.
2. The technique can be combined with other endoscopic intraventricular procedures like an ETV. A careful trajectory planning is mandatory.
3. Intraoperative hemorrhages are a frequent problem in tumor surgery. Almost in all cases, an intraoperative blurring of vision occurs. The surgeon should be prepared for this circumstance.
4. The position of the endoscope should remain stationary, when the bleeding occurs and a constant irrigation should be ensured all the time.
5. The management of intraoperative hemorrhage should be performed in an escalating technique starting with constant irrigation. The next steps should include coagulation, the option of mild compression, SCT and in the last consequence the application of the DFT.
6. Most of the hemorrhages can be stopped by constant irrigation.
7. The correct application of the DFT does not bear a high risk for ventricle collapse or postoperative subdural hematoma.
8. Postoperative surveillance on an intensive care unit is mandatory. An additional EVD could be placed.
9. Severe postoperative complications are rare. Fever is a common symptom after extensive irrigation.
10. The conversation rate to a microsurgical approach remains below 5% in our case series.

## Supplementary Information

Below is the link to the electronic supplementary material.

Supplementary file1 (MP4 433983 kb) Video. Examples of the various intraoperative hemorrhage types are demonstrated. Subsequently, the techniques to reobtain hemostasis such as compression, small chamber irrigation, and dry field are presented.

## Abbreviations

CSF: Cerebral spine fluid
ETV: Endoscopic third ventriculostomy
ICP: Intracranial pressure
EVD: External ventricular drainage
DFT: Dry field technique; SCT: small chamber technique
